# Acute metabolic responses to high‐intensity interval training in men with overweight or obesity: Does the exercise modality matter?

**DOI:** 10.1113/EP093045

**Published:** 2025-12-02

**Authors:** Annaëlle Couvert, Mélanie Rance, Eric Doré, Vincent Martin, Duane Beraud, Claire Morel, Bruno Pereira, Antonio Herbert Lancha, Nathalie Boisseau

**Affiliations:** ^1^ Laboratoire des Adaptations Métaboliques à l'Exercice en conditions Physiologiques et Pathologiques (AME2P) Université Clermont Auvergne Clermont‐Ferrand France; ^2^ Centre de Recherche en Nutrition Humaine – Auvergne – Rhône‐Alpes (CNRH‐AURA) Clermont‐Ferrand France; ^3^ Center of Resources Expertise and Performance in Sports (CREPS) Bellerive‐sur‐Allier France; ^4^ Institut Universitaire de France (IUF) Paris France; ^5^ CIC INSERM 1405/Plateforme d'Investigation Clinique CHU Gabriel Montpied Clermont‐Ferrand France; ^6^ Laboratory of Applied Nutrition and Metabolism, School of Physical Education and Sport University of São Paulo São Paulo Brazil

**Keywords:** energy intake, exercise modalities, fat oxidation, high intensity interval exercise, oxygen consumption

## Abstract

This study investigated the acute effects of two isoenergetic high‐intensity interval exercise (HIIE) sessions, running (HIIE‐RUN) and cycling (HIIE‐BIKE), on post‐exercise oxygen consumption (${\dot{V}}_{O_{2}}$), carbon dioxide production (${\dot{V}}_{CO_{2}}$), substrate oxidation and 24‐h energy intake (EI) in men with overweight or obesity. Twelve fasted men (44.4 ± 14.5 years; body mass index: 28.3 ± 1.9 kg m^−2^) completed both HIIE sessions. ${\dot{V}}_{O_{2}}$ and ${\dot{V}}_{CO_{2}}$ were measured before, during and after exercise, while substrate oxidation was calculated before and after exercise. The rate of perceived exertion was recorded during each exercise. Appetite was assessed throughout each session using a visual analogue scale (VAS) and EI was recorded via a 24‐h dietary questionnaire. Both exercise modalities resulted in similar energy expenditure (EE), but HIIE‐BIKE elicited a significantly higher respiratory exchange ratio (*P* = 0.002). No significant effect of exercise modality or time × modality interaction was observed for ${\dot{V}}_{O_{2}}$ and EE during the post‐exercise period. Fat oxidation was significantly increased during recovery compared with the pre‐exercise levels (*P* < 0.001), but did not differ between modalities. Appetite and 24‐h EI were unaffected by the exercise modality. In men with overweight or obesity, isoenergetic HIIE‐RUN and HIIE‐BIKE seem to induce comparable post‐exercise ${\dot{V}}_{O_{2}}$, EE and substrate oxidation during the 2‐h recovery period. Both modalities similarly promoted fat oxidation without specific dietary compensation observed.

## Abstract (highlights)

1. **What is the central question of this study?**
  Can the modality of isoenergetic high‐intensity interval exercise (running versus cycling) elicit distinct acute metabolic (e.g., post‐exercise fat oxidation, EPOC) and behavioural (e.g., appetite regulation, energy intake) responses that could differentially influence fat mass reduction and contribute to long‐term weight management in men with overweight or obesity?
2. **What is the main finding and its importance?**
  Isoenergetic high‐intensity interval exercise running and cycling seem to induce comparable metabolic and nutritional responses, indicating that the choice between these modalities can be guided by individual preferences and capabilities.

## 1 INTRODUCTION

Overweight and obesity, which are characterized by excessive fat mass (FM) accumulation, affect more than 2 billion adults worldwide and their prevalence continues to rise (Ng et al., 2025). Excess FM and its associated metabolic disturbances increase the risk of cardiovascular diseases, type 2 diabetes and various cancers (Powell‐Wiley et al., 2021). Besides total FM, abdominal FM, particularly visceral adipose tissue (VAT), is strongly linked to adverse health outcomes (Palmer & Clegg, 2015). Accordingly, reducing both total and abdominal FM may help to mitigate these health risks (Ross et al., 2020).

Regular exercise is a key strategy for addressing overweight and obesity by reducing adipose tissue (Bellicha et al., 2021). High‐intensity interval training (HIIT), which consists of repeated bouts of high‐intensity efforts interspersed with short recovery periods (Coates et al., 2023), is recognized as a time‐efficient strategy to reduce adiposity (Maillard et al., 2018; Wewege et al., 2017). However, the HIIT modality (i.e., running vs. cycling) may differentially affect body composition. In a meta‐analysis including adults with normal weight and overweight or obesity, Maillard et al. (2018) found that running was more effective than cycling in reducing total FM and visceral adipose tissue, while cycling was more beneficial for decreasing total abdominal FM (Maillard et al., 2018). However, this meta‐analysis compared studies with highly heterogeneous HIIT protocols, making its conclusions debatable. A more recent meta‐analysis by Khodadadi et al. (2023) also reported greater reductions in body fat percentage with HIIT running compared to HIIT cycling, although the analysed studies again lacked standardization, particularly in terms of energy expenditure (EE) (Khodadadi et al., 2023).

To address this limitation, our team recently conducted a study that compared two isoenergetic 3‐month HIIT programmes (running vs. cycling) in men with overweight or moderate obesity. Both exercise modalities led to significant reductions in total, abdominal and visceral FM, with a slightly greater decrease in abdominal fat observed in the running group. However, no significant difference was found between the two modalities regarding total FM reduction (Couvert et al., 2024). While both modalities elicited comparable chronic effects after 3 months of training (3 sessions per week), some evidence suggests that the acute metabolic responses to high‐intensity interval exercise (HIIE) may differ between running and cycling. These differences could involve excess post‐exercise oxygen consumption (EPOC), potentially affecting post‐exercise carbohydrate and fat oxidation. Over time, a greater reliance on fat oxidation during the recovery period could contribute to long‐term FM reduction. The specific characteristics of running and cycling, such as the lower muscle mass involved in cycling (Millet, 2009), the predominance of concentric vs. eccentric muscle contractions affecting muscle damage (Bijker et al., 2002), and variations in catecholamine production at the same relative intensity (Zouhal et al., 2008), may contribute to potential disparities in EPOC and fat oxidation during recovery. Cunha et al. reported that after isoenergetic continuous (400 kcal) or intermittent exercise bouts (2 × 200 kcal) at 75% of oxygen uptake reserve, EPOC was higher in runners than cyclists during a 60‐min recovery (Cunha et al., 2016). In contrast, Townsend et al. found no difference in EPOC following sprint interval training sessions in cycling and running (Townsend et al., 2014). To date, no study has directly compared the effects of isoenergetic cycling and running HIIE on post‐exercise oxygen consumption and fat oxidation, despite their potential relevance for long‐term FM reduction.

Besides the metabolic responses, exercise may also influence body composition through its acute effects on appetite and energy intake (EI), which should be considered a potential confounding factor when comparing HIIE modalities. Similar to other high‐intensity exercises (>70% ${\dot{V}}_{O_{2}\max}$), acute HIIE transiently alters the blood concentration of appetite‐related hormones (Hu et al., 2023), reduces appetite perception assessed with a visual analogue scale (VAS) (Hu et al., 2022), and lowers ad libitum post‐exercise EI (Sim et al., 2015). These effects have been widely studied in comparisons between acute HIIE and moderate‐intensity continuous exercise (Dupuit et al., 2020; Hu et al., 2022; Matos et al., 2018). Conversely, fewer studies have directly compared different HIIE modalities, such as running and cycling. A meta‐analysis by Schubert et al. found that exercise has a small to moderate impact on appetite hormones, and the exercise modality does not appear to influence these hormonal responses (Schubert et al., 2014). To date, evidence directly comparing the effects of high‐intensity running and cycling on appetite regulation is extremely limited. Only one study has assessed perceived hunger (VAS) and plasma acylated ghrelin and found no significant difference between modalities in young, normal‐weight men (Wasse et al., 2013).

Based on these considerations, the aim of the present pilot study was to compare oxygen consumption (${\dot{V}}_{O_{2}}$), EE, substrate oxidation (carbohydrate and fat), and appetite during the 2‐h recovery period, as well as EI over the subsequent 24 h, following acute isoenergetic HIIE sessions of running and cycling. The overarching objective was to determine whether one modality induces greater EE or fat oxidation, or a stronger reduction in appetite and EI, thereby providing insights into their potential contribution to weight and FM management in individuals with overweight or obesity.

## 2 METHODS

This protocol was implemented within the usual care framework for men with overweight/obesity at the Resource, Expertise and Sports Performance Center (CREPS) Auvergne Rhône‐Alpes, Vichy, France, between 2023 and 2024. Ethical approval was obtained from a French Ethics Committee for Research in Sports Sciences (IRB number: 00012476‐2022‐22‐09‐199), and all procedures complied with the principles of the *Declaration of Helsinki*. All men underwent a medical examination before starting the protocol (pre‐participation health screening performed by a sports physician). After receiving detailed information on the study objectives and protocol, each participant signed a written informed consent.

### 2.1 Participants

Twelve men (aged 18–65 years) with overweight or class I obesity (body mass index (BMI) 25–35 kg/m^2^) were recruited from the Sports Health Medical Center at the CREPS of Vichy. All participants completed the full protocol. Exclusion criteria were beta‐blocker use, diabetes, medical contraindications to vigorous exercise, joint pain, chronic arterial, respiratory, cardiovascular or endocrine diseases, and weight‐loss drug use. All had low physical activity levels (<150 min per week) according to the World Health Organization guidelines, assessed via the Global Physical Activity Questionnaire (Cleland et al., 2014).

The sample size was calculated a priori using EPOC data from Cunha et al. (2016) who compared continuous and intermittent aerobic running and cycling in healthy young men. Their findings indicated that EPOC was higher (by 37%) after isoenergetic sessions (400 kcal) of continuous or intermittent running than cycling (*P* < 0.001). Based on these results, a power analysis suggested that at least 10 participants would be required to detect a moderate effect size between exercise conditions (α = 0.05, β = 0.10). To account for potential dropouts, the sample size was increased to 12 men for this pilot study.

### 2.2 Overview of study design

**FIGURE 1:**
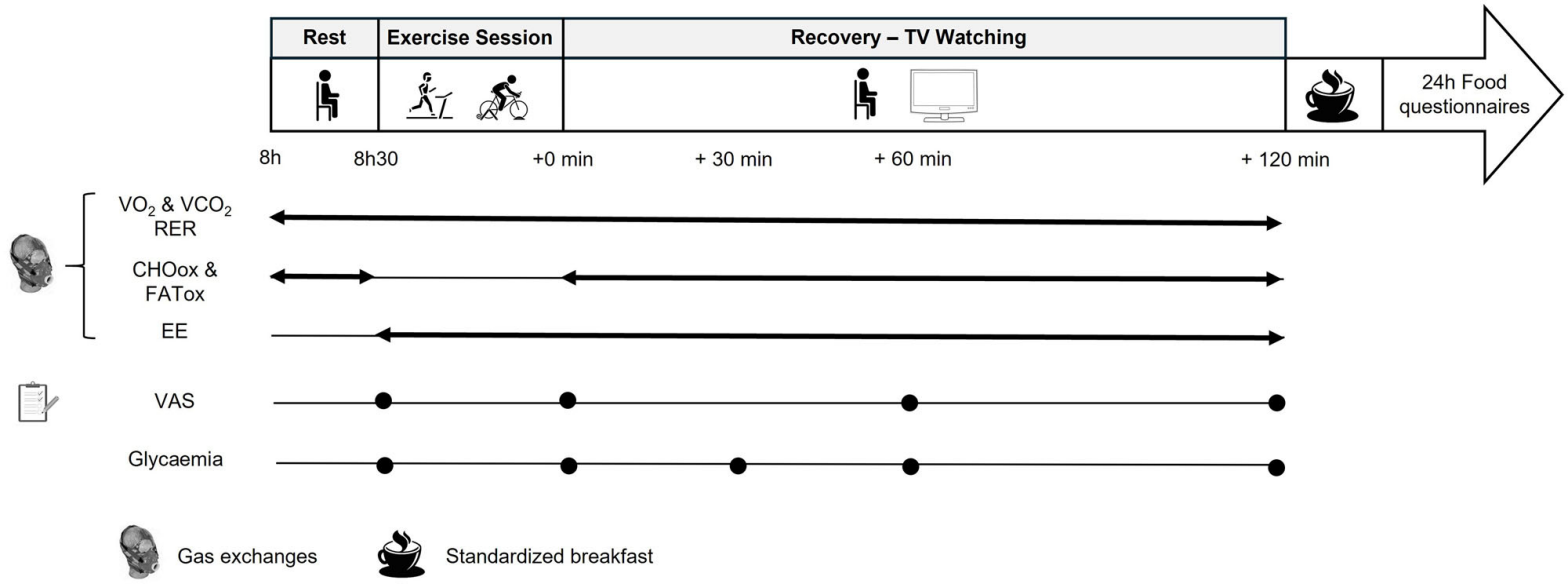
Experimental Design. CHOox, carbohydrate oxidation (g·min^−1^); EE, energy expenditure (kcal); FATox, fat oxidation (g·min^−1^); RER, respiratory exchange ratio; VAS, visual analogue scale (appetite perception); RPE, rate of perceived exertion.

The protocol comprised three visits. The first one (inclusion visit) consisted of a medical exam followed by a familiarization with both ergometers. Visits 2 and 3 corresponded to the exercise sessions (Figure 1). For the exercise sessions, participants arrived at 08.00 h after ≥10 h of fasting. After recording perceived appetite (VAS) and fasting glycaemia (Accu‐Chek), participants rested for 10 min. Gas exchanges (${\dot{V}}_{O_{2}}$, ${\dot{V}}_{CO_{2}}$) were then measured for 15 min (Metamax 3B, MTraining, Ecole‐Valentin, France) before the exercise session. Participants were then positioned on the bike or treadmill to perform the exercise session. The rate of perceived exertion (RPE), appetite perception (VAS) and blood glucose (monitored to detect potential hypoglycaemia) were measured at regular intervals during the exercise and throughout the subsequent 2‐h recovery period. During the recovery period, participants remained seated and watched TV, while gas exchanges were continuously recorded. They subsequently consumed a standardized breakfast before leaving the facility. At home, they completed a 24‐h dietary intake questionnaire until the next day's breakfast.

### 2.3 Anthropometric and body composition measurements

Anthropometric and body composition measurements were carried out before each exercise session to verify that no significant variation occurred between sessions. Body weight was measured to the nearest 0.1 kg using a calibrating scale (Balance Seca 709, Hamburg, France) under fasting conditions, with participants wearing only underwear. Height was measured to the nearest 0.5 cm with a wall‐mounted stadiometer and BMI was calculated as body weight (kg) divided by the square of height (m^2^). Waist circumference (cm) was measured midway between the last rib and the upper iliac crest (cm), and hip circumference (cm) was measured as the largest circumference around the buttocks, in standing position with a measuring tape. Body fat percentage was estimated by the same experimenter using skinfold thickness measurements at four sites: biceps, triceps, subscapular and supra‐iliac regions (Durnin & Rahaman, 1967).

### 2.4 Exercise sessions

Each participant completed two exercise sessions: HIIE running (HIIE‐RUN) and HIIE cycling (HIIE‐BIKE). The sessions were conducted in a randomized counterbalanced design with a 7‐ to 14‐day interval to allow for recovery. Randomization assigned six participants to begin with the HIIE‐BIKE session and six with the HIIE‐RUN. On the day preceding each exercise session, participants were instructed to have the same dinner (their choice) at the same time, refrain from vigorous exercise for 48 h, and abstain from coffee, alcohol and tobacco for 24 h. The two exercise sessions were designed to be isoenergetic. EE during each exercise session was assessed from the oxygen consumption measured breath‐by‐breath with a portable Metamax 3B automated gas analysis system (Matsport, France). To ensure that both modalities were isoenergetic, EE was continuously monitored, and the second session was stopped once it matched the EE of the first session. The mean EE for each session was 296.8 ± 40.9 kcal for HIIE‐BIKE and 294.5 ± 45.1 kcal for HIIE‐RUN and was obtained by 10 intervals for the first modality tested. Maximum heart rate (HR_max_) was estimated using the Tanaka's formula (Tanaka et al., 2001): HR_max_ = 208 – (0.7 × age). The two modalities were as follows:

**HIIE‐BIKE session**. After a 5‐min warm‐up at 55% HR_max_ on the bike (Cyclus2, RBM Elektronik‐Automation GmbH, Leipzig, Germany), participants performed 9–11 cycles of 45 s of high intensity cycling followed by a 90 s active recovery period. The power outputs were individually predetermined and corresponded to 80–85% of HR_max_ during the high intensity phases and 50–55% of HR_max_ during the recovery phase. Each participant's resistance, pedal cadence (60–70 rpm), heart rate (A360, Polar, Kempele, Finland; beat min^−1^) and power (watts) were controlled to reach the expected intensity.
**HIIE‐RUN session**. After a 5‐min warm‐up on the treadmill at 55% HR_max_ (Quasar® h/p/cosmos, Nussdorf‐Traunstein, Germany), participants performed 9–11 cycles of 45 s of high intensity phases followed by a 90 s active recovery period. The speed levels to be achieved on the treadmill were individually predetermined and corresponded to 80–85% of HR_max_ during the high intensity phases and 50–55% of HR_max_ during the recovery phase. The treadmill gradient was set to 1% to simulate real conditions (Jones & Doust, 1996). Each participant's heart rate (A360, Polar, Kempele, Finland; beats min^−1^) and speed (km h^−1^) were controlled to reach the expected intensity.

RPE was recorded using the CR‐10 Scale (Borg, 1982) after completing 33% and 66% of repetitions, as well as at the exercise end.

For both modalities, the final low‐intensity interval (90 s) acted as a brief active cool‐down period before moving to the 2‐h seated recovery period.

### 2.5 Indirect calorimetry

The experiment was conducted in a ventilated room maintained at a temperature of 19–21°C. Gas exchange measurements were continuously monitored throughout the sessions using a Metamax 3B apparatus (Matsport) and data recorded.

EE (kcal) was calculated at rest, during exercise and during the recovery period using the mean ${\dot{V}}_{O_{2}}$ and the thermal coefficient of O_2_ corresponding to the recorded respiratory exchange ratio (RER): EE (kcal) = ${\dot{V}}_{O_{2}}$ (L min^−1^) × time (min) × O_2_ coefficient.

During exercise, EE was provided in real time by the MetaMax system. EPOC was determined as the area under the ${\dot{V}}_{O_{2}}$ curve during the recovery phase, minus the area under the ${\dot{V}}_{O_{2}}$ curve at rest prior to exercise. The relative contribution of carbohydrate and fat was estimated at rest and during the 2‐h recovery period using the following equations:

$$\mathrm{Fat}\;\left(\%\right)=\left(1-\mathrm{RER}\right)/\left(0.3\right)\times 100$$

$$\mathrm{Carbohydrate}\;\left(\%\right)=100-\mathrm{Fat}\;\left(\%\right).$$

Carbohydrate oxidation (CHOox) and fat oxidation (FATox) (g min^−1^) were calculated using the Perronet and Massicotte equations (Péronnet & Massicotte, 1991):

$$\mathrm{CHOox}\left(g\;\min^{-1}\right)=\left(4.585\times {\dot{V}}_{CO_{2}}\right)-\left(3.22255\times {\dot{V}}_{O_{2}}\right)$$

$$\mathrm{FATox}\left(g\;\min^{-1}\right)=\left(1.6946\times {\dot{V}}_{O_{2}}\right)-\left(1.7012\times {\dot{V}}_{CO_{2}}\right)$$

During exercise, data were averaged over the 5 min preceding each time point (33%, 66% and 100%) to ensure consistency with the 5‐min periods used during the pre‐exercise and post‐exercise recovery.

### 2.6 Glycaemia

To mitigate the risk of hypoglycaemia, blood glucose concentration was measured from fingertip blood using an Accu‐Chek Performa meter (Roche Diabetes Care, France) before exercise, immediately post‐exercise, and at 30 min, 1 and 2 h during recovery (Figure 1).

### 2.7 Appetite rating and breakfast composition

Appetite perception was assessed at multiple time points, before exercise, immediately post‐exercise, and during recovery (at 60 and 120 min), using a validated 100‐mm VAS (Flint et al., 2000). This scale includes four questions anchored with words that represent the two extreme states of desire to eat, hunger, fullness and prospective food consumption. The average appetite score was calculated using the following formula: Appetite score = (desire to eat + hunger + (100 − fullness) + prospective consumption)/4.

Following the recovery period, participants were instructed to consume a standardized breakfast within 15 min. Each participant selected their meal, ensuring consistency across both sessions. The breakfast included bread, butter, fruit and/or orange juice, dairy products, and tea or coffee.

### 2.8 Dietary assessment

Participants were asked to complete a food diary detailing the type and quantity of all foods consumed from lunch following the exercise session until breakfast the next morning. Participants weighed their food (specifying whether raw or cooked) or estimated portion sizes (e.g., teaspoons for oil). For packaged products, they reported the brand and product reference. Total EI (kcal) and macronutrient composition were analysed using the Nutrilog® software (Marans, France).

### 2.9 Statistical analysis

All statistical analyses were carried out with STATISTICA version 12.00 (StatSoft Inc., Tulsa, OK, USA).

Concerning the study outcomes, post‐exercise EE (total ${\dot{V}}_{O_{2}}$ during the 2‐h recovery) was defined as the primary endpoint and FATox a co‐primary endpoint. Secondary outcomes included EPOC, appetite (VAS) and 24‐h EI. The power calculation was based on EPOC (Cunha et al., 2016) due to the lack of comparable data for ${\dot{V}}_{O_{2}}$ or FATox in overweight/obese populations.

Data are presented as means ± SD. The data normal distribution was tested using the Shapiro–Wilk test, and the homogeneity of variance with the *F*‐test. Data were log‐transformed, when appropriate, before statistical analyses. For calorimetric analysis, a two‐way repeated‐measures ANOVA was used to assess changes over time and between modalities, identifying time, modality and interaction effects. The 95% confidence intervals and effect sizes from the repeated‐measures ANOVA are provided in the Supporting information. When a significant effect was detected, *post hoc* multiple comparisons were performed using Tukey's HSD test. To compare two time points or periods within the same modality or between modalities, a paired‐sample non‐parametric test was used (Wilcoxon test). Appetite scores and sub‐scores were expressed as the area under the curve (AUC), calculated using the trapezoidal method, and analysed with the Wilcoxon test. EI, including macronutrient distribution, was also analysed using the same test. EPOC was expressed as the net AUC of ${\dot{V}}_{O_{2}}$ during recovery, after subtracting the mean resting ${\dot{V}}_{O_{2}}$ value (mean resting ${\dot{V}}_{O_{2}}$ value (L min^−1^) × 120). Differences with a *P‐*value ≤ 0.05 were considered significant.

## 3 RESULTS

### 3.1 Participants’ characteristics

All twelve participants completed the entire protocol (no screen failure or dropout). Their mean age, weight, BMI, waist circumference and body FM percentage were 44.4 ± 14.5 years, 86.6 ± 5.1 kg, 28.3 ± 1.9 kg m^−2^, 95.4 ± 5.8 cm and 26.2 ± 2.0%, respectively. The mean differences in body weight and body fat percentage (skinfold thickness) between sessions were −0.11 ± 1.02 kg and −0.43 ± 1.02%, respectively. These differences can be considered negligible and fall within the expected range for such a short time interval.

### 3.2 Exercise sessions

No adverse events, such as pain or hypoglycaemia, were reported during both exercise sessions. The mean HIIE duration was similar between modalities: 27.7 ± 2.3 min (10.1 ± 1.0 intervals) for HIIE‐RUN and 27.3 ± 1.5 min (9.9 ± 0.7 intervals) for HIIE‐BIKE. Heart rate monitoring during the acceleration and deceleration phases indicated similar exercise intensity, with mean HR_max_ values of 77.2 ± 5.1% for HIIE‐RUN and 79.3 ± 4.5% for HIIE‐BIKE.

### 3.3 Oxygen consumption

**FIGURE 2:**
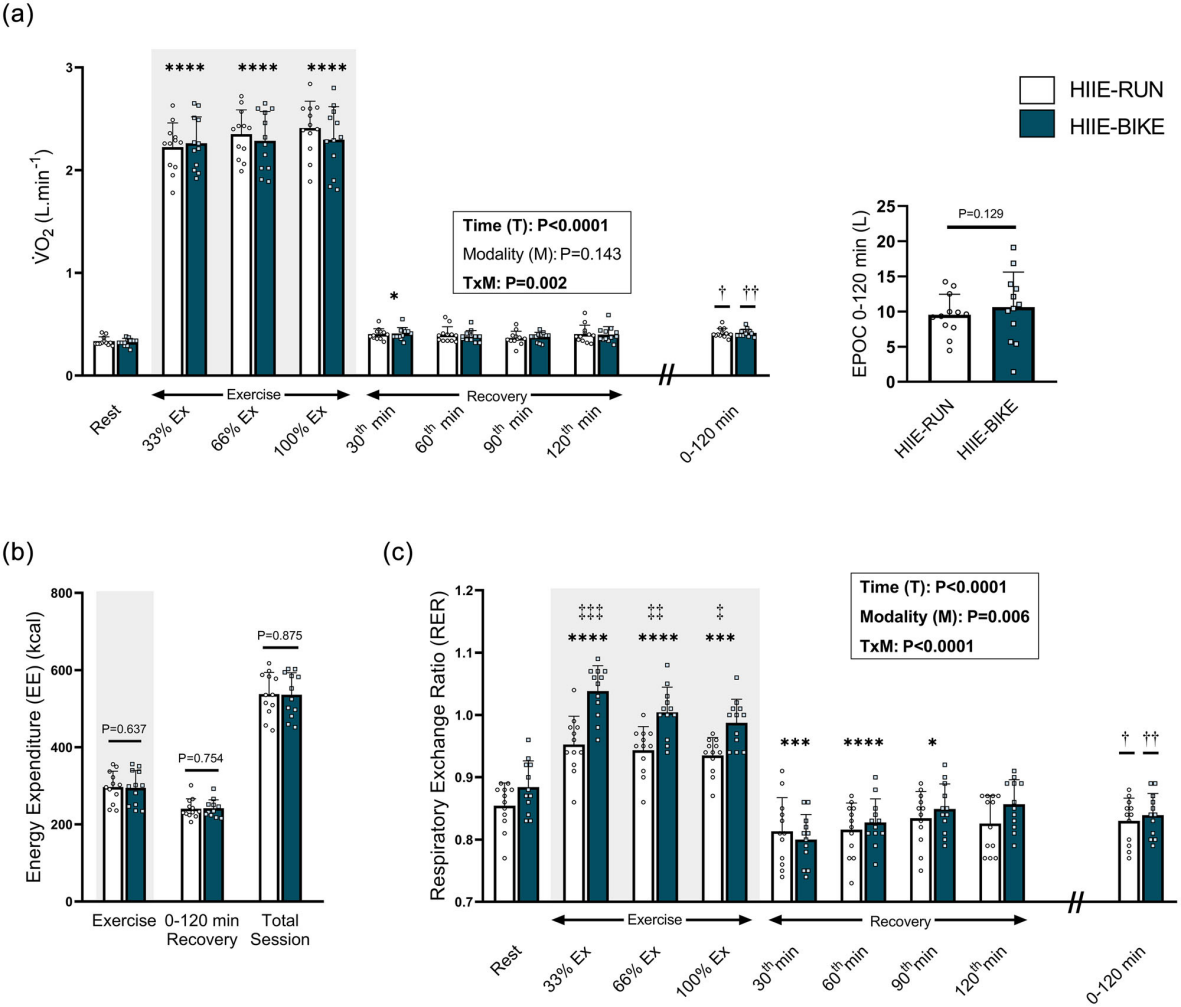
Oxygen consumption (${\dot{V}}_{O_{2}}$) (a), energy expenditure (EE) (b), and respiratory exchange ratio (RER) (c) during exercise: total values and at specific time points during recovery. Values are means ± SD, *n* = 12. *P < 0.05, **P < 0.01, ***P < 0.001, ****P < 0.0001, time effect vs. rest; ‡*P* < 0.05, ‡‡*P* < 0.01, ‡‡‡*P* < 0.001, time × modality effect; †*P* < 0.05, ††*P* < 0.01, mean recovery values vs. resting values (Wilcoxon test).

${\dot{V}}_{O_{2}}$ increased during exercise compared with rest, regardless of the modality (all *P* < 0.0001) (Figure 2a). ${\dot{V}}_{O_{2}}$ was not significantly different between modalities at the first, second and third stage of exercise (33%, 66% and 100%) (all *P* > 0.284). EPOC over the 2‐h recovery period, represented by the net AUC, did not differ between conditions (*P* = 0.129). Precisely, no significant differences in ${\dot{V}}_{O_{2}}$ were observed at any time point (30, 60, 90 and 120 min of recovery) (all *P* > 0.989). However, ${\dot{V}}_{O_{2}}$ was significantly higher at 30 min post‐exercise compared with rest values (*P* = 0.034).

### 3.4 Energy expenditure

As expected, based on the protocol design, EE during exercise did not differ between modalities (297 ± 41 kcal for HIIE‐RUN vs. 294 ± 45 kcal for HIIE‐BIKE, *P* = 0.637). During the recovery period, similar EE values were observed for both modalities (241 ± 25 vs. 242 ± 22 kcal, *P* = 0.754 for HIIE‐RUN and HIIE‐BIKE, respectively) (Figure 2b). Consequently, the total EE per session was similar between conditions (538 ± 56 vs. 536 ± 57 kcal for HIIE‐RUN and HIIE‐BIKE, respectively; *P* = 0.875).

### 3.5 Respiratory exchange ratio

Acute HIIE induced a significant increase in RER from resting levels, regardless of the exercise modality (all *P* < 0.001) (Figure 2c). However, RER was higher during the HIIE‐BIKE than HIIE‐RUN session at all exercise stages (all *P* < 0.044; mean RER: 0.98 ± 0.03 vs. 0.91 ± 0.03, *P* = 0.002), suggesting a greater reliance on CHOox and/or increased hyperventilation. RER values during recovery did not differ between modalities at any measured time point (all *P* > 0.890). However, RER was significantly lower than resting values at 30, 60 and 90 min post‐exercise (all *P* < 0.012). The mean RER over the 2‐h recovery period was similar between HIIE‐RUN and HIIE‐BIKE (0.83 ± 0.04 and 0.84 ± 0.03; *P* = 0.388), but significantly lower than the resting values for both modalities (*P* = 0.012 and *P* = 0.002, respectively).

### 3.6 Substrate oxidation (% and g·min^−1^)

**FIGURE 3:**
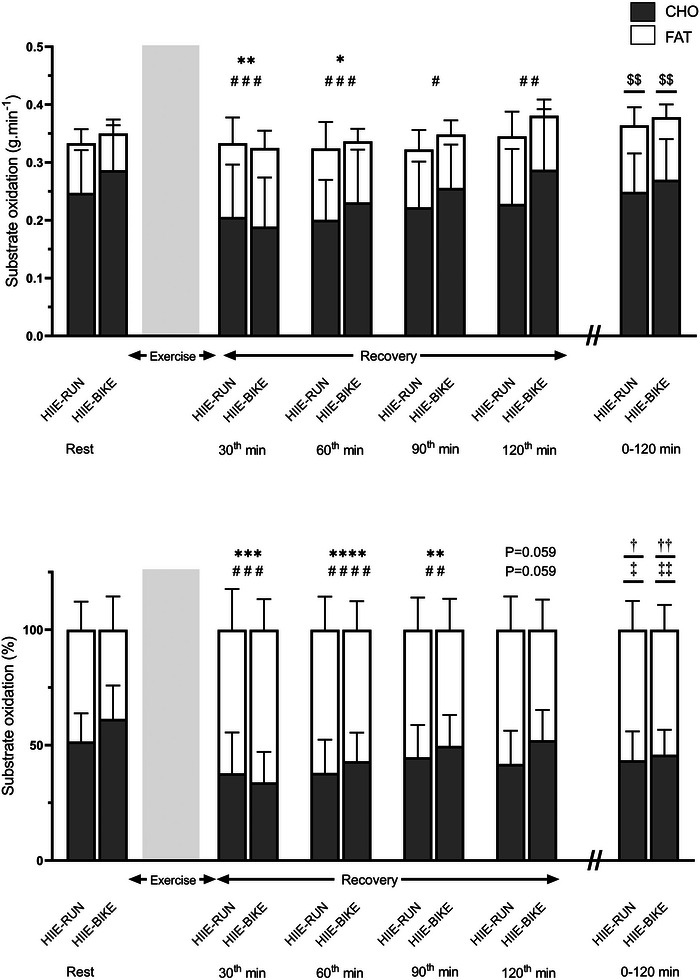
Substrate oxidation (g·min^−1^ and %). Values are means ± SD, *n* = 12. *P < 0.05, **P < 0.01, ***P < 0.001, ****: *P* < 0.0001, time effect for CHO oxidation vs. rest.; #*P* < 0.05, ##*P* < 0.01, ###*P* < 0.001, ####*P* < 0.0001, time effect for FAT oxidation vs. rest. †*P* < 0.05, ††*P* < 0.01, Mean CHO oxidation values during recovery vs. values at rest (Wilcoxon test). ‡*P* < 0.05, ‡‡*P* < 0.01, Mean fat oxidation values during recovery vs. values at rest (Wilcoxon test).

RER values measured during the exercise sessions occasionally exceeded 1.0, precluding the reliable estimation of CHOox and FATox rates because the standard stoichiometric equations become inaccurate in conditions of hyperventilation induced by high‐intensity exercise. Throughout the recovery period, CHOox and FATox rates and their respective contributions were similar between HIIE‐BIKE and HIIE‐RUN at all measured time points (30, 60, 90 and 120 min of recovery) (Figure 3). Overall, there was no significant difference in the mean CHOox and FATox between modalities during recovery, when expressed as percentages (*P* = 0.531 and *P* = 0.531, respectively) or total grams oxidized (*P* = 0.209 and *P* = 0.388, respectively). At all recovery time points, FATox rates were significantly higher than at rest (all *P* < 0.027) (Figure 3). FATox expressed as a percentage of EE was significantly higher at 30, 60 and 90 min post‐exercise (all *P* < 0.006), with a trend toward significance at 120 min (*P* = 0.059).

### 3.7 Rate of perceived exertion

**FIGURE 4:**
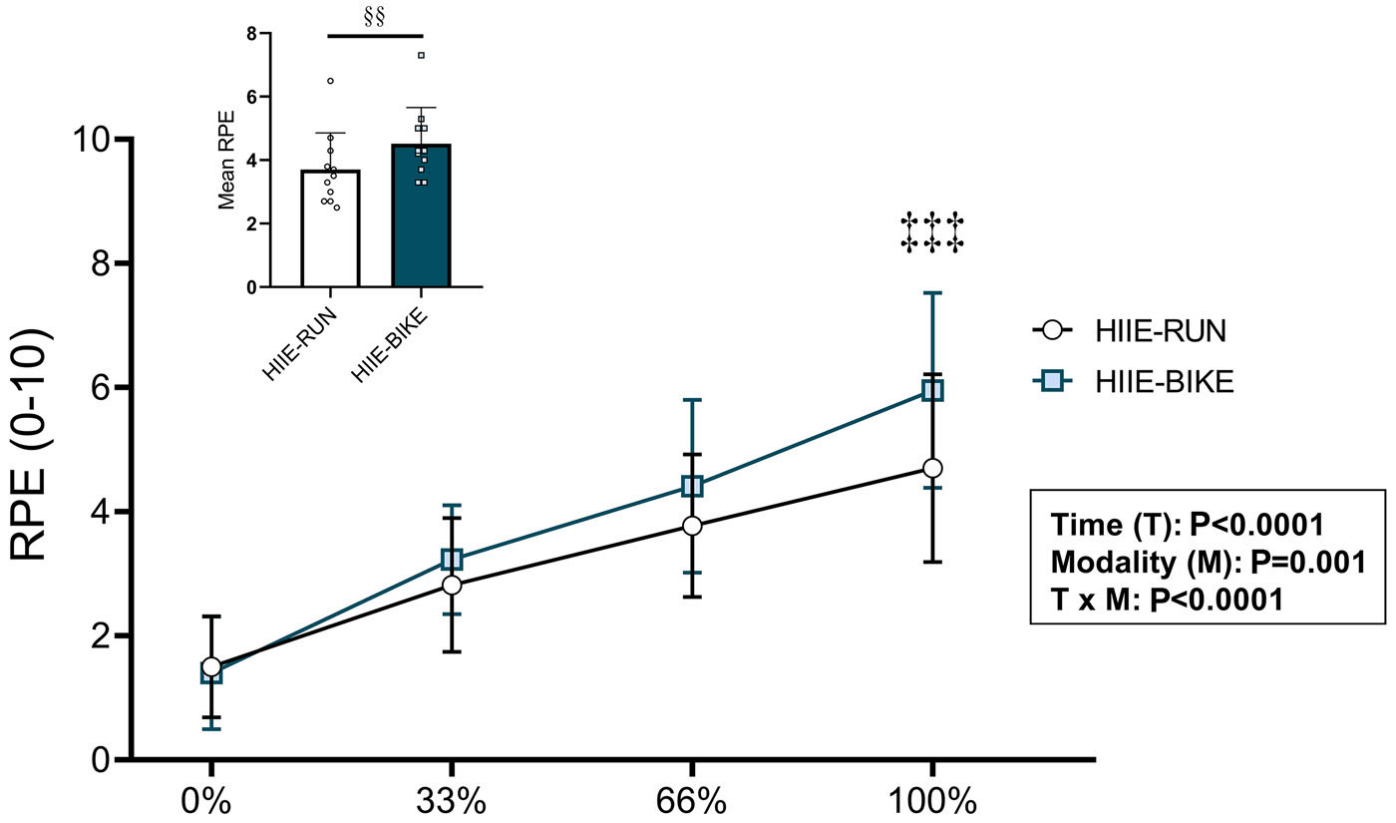
RPE at rest (baseline) and at 33%, 66% and 100% of exercise completion. Values are means ± SD, *n* = 12. ‡‡‡*P* < 0.001, time × modality effect. §§*P* < 0.01, significant difference between HIIE‐RUN and HIIE‐BIKE. RPE, rate of perceived exertion.

RPE increased progressively at 33%, 66% and 100% of the exercise session for both modalities (time effect, *P* < 0.0001), with significantly higher values at the end of the cycling than running session (*P* = 0.0001) (Figure 4). A significant modality effect was also observed, indicating overall higher RPE values for HIIE‐BIKE than for HIIE‐RUN (*P* = 0.001). Similarly, the mean RPE across the entire exercise session was significantly higher in the cycling condition (*P* = 0.003) (inset in Figure 4).

### 3.8 Subjective appetite ratings

**FIGURE 5:**
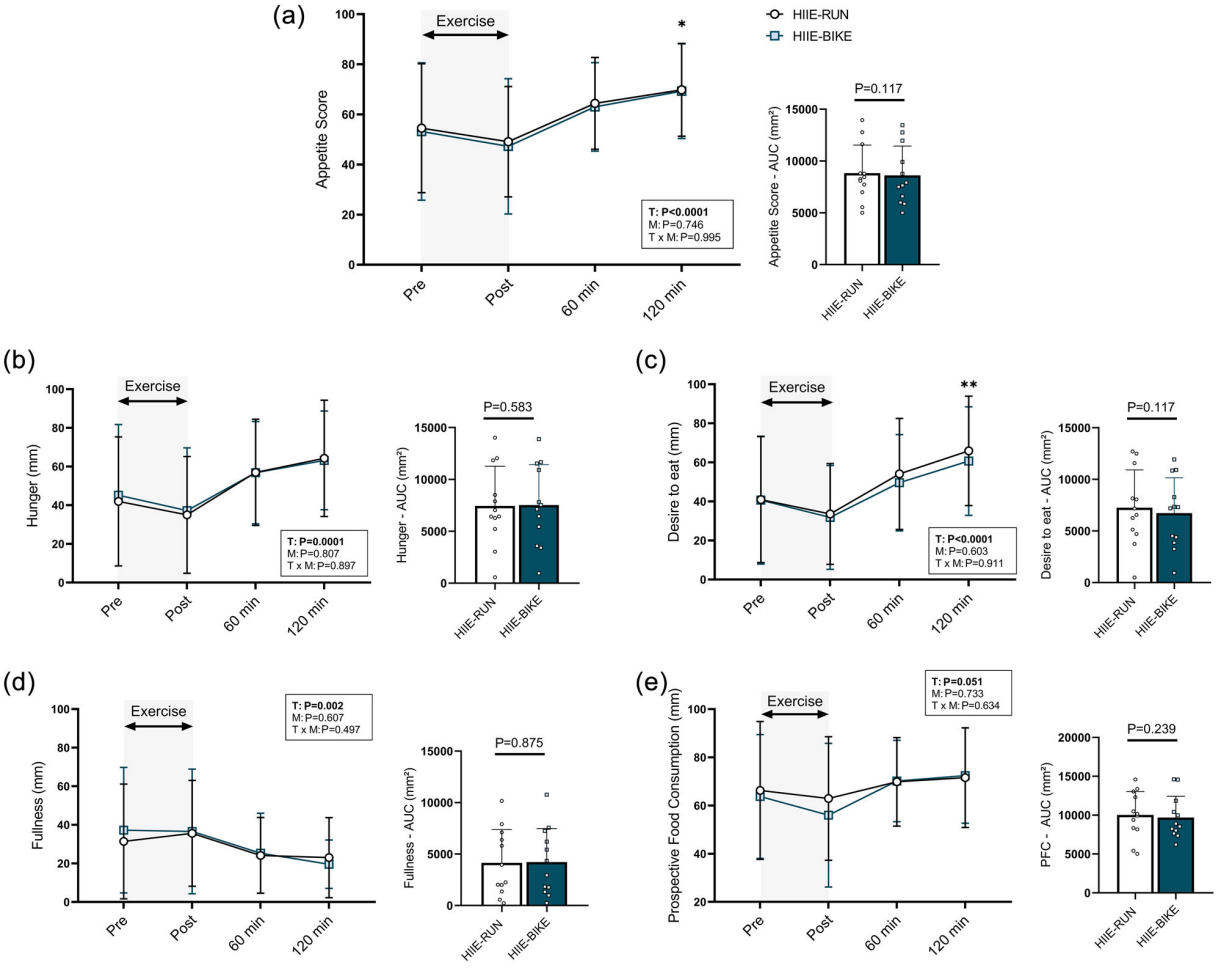
(a) Appetite score before the exercise session (Pre), immediately post‐exercise (Post), and at 60 and 120 min of recovery. (b) VAS score for hunger, (c) desire to eat, (d) fullness, (e) prospective food consumption, before exercise (Pre), immediately post‐exercise (Post), and at 60 and 120 min of recovery. Values are means ± SD, *n* = 12. *P < 0.05, **P < 0.01 vs. pre‐exercise (Pre).

Compared to pre‐exercise, perceived appetite scores (VAS) showed a significant time effect for both modalities (*P* < 0.0001), with higher values at 120‐min post‐exercise (*P* = 0.041). However, no significant difference was found between running and cycling during the recovery period (Figure 5a). All four appetite sub‐scores (desire to eat, hunger, fullness and prospective food consumption) were not different between modalities (Figure 5b–e). When analysed as AUC values, neither the overall perceived appetite score nor any of the appetite sub‐scores differed between HIIE‐BIKE and HIIE‐RUN (Figure 5).

### 3.9 Post‐exercise energy intake

The meal consumed the night before each exercise session was self‐selected and replicated before the second session. The 24‐h post‐exercise period began immediately after the standardized breakfast following the exercise session and extended until the same time the next day, covering lunch, dinner, breakfast and snacks. EI (kcal) and macronutrient distribution (carbohydrate, fat and protein) are detailed in Table 1, without significant differences between modalities.

**TABLE 1:** Acute 24‐h energy and macronutrient intake after the HIIE‐RUN and HIIE‐BIKE sessions.

	HIIE‐RUN	HIIE‐BIKE	P (modality effect)
Energy intake (kcal)	1931 ± 803	2043 ± 450	0.583
Lunch	723 ± 427	703 ± 375	0.386
Snack	153 ± 210	113 ± 187	0.575
Dinner	748 ± 332	909 ± 360	0.308
Breakfast	308 ± 202	318 ± 273	0.424
Macronutrient intake (% 24 h)			
Carbohydrate	46.2 ± 9.7	40.8 ± 7.2	0.347
Fat	32.7 ± 7.7	37.5 ± 10.1	0.327
Protein	17.2 ± 4.8	17.7 ± 5.1	0.754
Alcohol	0.9 ± 1.8	1.6 ± 2.9	0.345

*Note*: Data are presented as means ± SD with *n* = 12 subjects.

## 4 DISCUSSION

The aim of this pilot study was to investigate the acute effects of HIIE performed by running or cycling on factors associated with FM loss, including ${\dot{V}}_{O_{2}}$, EE, substrate oxidation, appetite and 24‐h EI. Although cycling elicited significantly higher RER and RPE than running, post‐exercise ${\dot{V}}_{O_{2}}$, EE, substrate oxidation, appetite perception and 24‐h EI were not different between modalities.

Two meta‐analyses (Khodadadi et al., 2023; Maillard et al., 2018) showed that HIIT programmes can lead to different levels of FM loss depending on whether they are performed through running or cycling. However, the studies included in these analyses did not specifically target individuals with overweight or obesity, and the protocols were not necessarily isoenergetic. To address this limitation, our research team recently conducted a 3‐month isoenergetic HIIT intervention, where running and cycling sessions were standardized to ensure equivalent EE in men with overweight and obesity (Couvert et al., 2024). Our findings demonstrated that both modalities were effective in reducing total FM, without significant differences between them, except for greater abdominal FM loss in the running group (Couvert et al., 2024). To further investigate these results, the present study compared key factors that may influence FM loss following an acute HIIE session, including EPOC, post‐exercise CHOox and FATox rates, as well as potential appetite modulation and dietary compensation over the next 24 h. EPOC is a well‐established response to high‐intensity exercise that occurs primarily within the first hour of recovery (Børsheim & Bahr, 2003; Moniz et al., 2020) and contributes to the total EE of the session. However, the distinct biomechanical and physiological characteristics of running and cycling suggest that EPOC responses may differ between these modalities. Compared to cycling, running recruits a larger muscle mass (Millet, 2009) and involves an eccentric component, which may result in greater muscle damage (Bijker et al., 2002). Exercise‐induced muscle damage can increase the resting EE in the following day (Hunter et al., 2017). Collectively, these factors suggest that post‐exercise ${\dot{V}}_{O_{2}}$ could be higher after an acute HIIE session performed by running rather than cycling, although no study compared standardized isoenergetic sessions.

Cunha et al. (2016) compared acute bouts of continuous exercise (running vs. cycling) with an EE of 400 kcal and intermittent bouts split into 2 × 200 kcal both performed at 75% of oxygen uptake reserve. They reported a slight increase in EPOC (+2.2 L) during the 60‐min recovery period following running compared with cycling (Cunha et al., 2016). However, this finding was not replicated by other studies that compared acute running and cycling sprint interval exercise (Townsend et al., 2014) or single 1‐min bouts (Scott et al., 2006). In the present study on the effects of acute isoenergetic running and cycling HIIE, ${\dot{V}}_{O_{2}}$ did not differ significantly between modalities during exercise and throughout the 2‐h recovery period. Therefore, when sessions are isoenergetic, both running and cycling have a similar effect on the 2‐h post‐exercise ${\dot{V}}_{O_{2}}$ and total EE (538 ± 56 kcal for HIIE‐RUN and 536 ± 57 kcal for HIIE‐BIKE).

This result was observed despite higher RER values during cycling HIIE than running HIIE. Previous studies also reported higher RER during cycling than running, both during submaximal exercise and across a wide range of intensities up to maximal effort (Achten et al., 2003; Chenevière et al., 2010; Lafortuna et al., 2010). Additionally, plasma lactate concentrations are consistently higher during cycling than running when exercise is performed at equivalent percentages of ${\dot{V}}_{O_{2}\max}$ or maximal workload (Achten et al., 2003; Capostagno & Bosch, 2010; Chenevière et al., 2010; Lafortuna et al., 2010; Millet, 2009). Comparable differences also are observed when intensity is matched using the heart rate (Kodesh et al., 2024), reflecting a greater reliance on glycolytic metabolism during cycling than running (Millet, 2009; Scott et al., 2006). Several physiological mechanisms have been proposed to explain these differences. First, the smaller muscle mass recruited during cycling may increase the metabolic demand per unit of active tissue (Lafortuna et al., 2010). Second, the greater intramuscular tension required during cycling, partly due to the lack of elastic energy restitution typically provided by eccentric muscle actions in running, may lead to increased recruitment of type II fibres (Carter et al., 2000). Moreover, in our study, intensity was determined as a percentage of the estimated HR_max_. However, in untrained individuals, both HR_max_ and ${\dot{V}}_{O_{2}\max}$ are typically higher during an incremental treadmill test than during a cycling test (Cunha et al., 2016; Millet, 2009). Therefore, we hypothesize that in our study, achieving the same percentage of estimated HR_max_ (75.2 ± 3.5% in cycling and 73.0 ± 4.4% in running) actually corresponded to a higher relative intensity in cycling than running (Abrantes et al., 2012). This is further supported by the higher RPE reported by participants during cycling HIIE. Increased perceived exertion is commonly observed during cycling than running, reinforcing the idea that cycling is perceived as more demanding than running for a given heart rate (Kodesh et al., 2024). Altogether, these findings indicate that the method used to set the exercise intensity must be considered carefully because it may partly explain the result variability observed among studies. However, despite these modality‐specific differences in RER observed during the exercise bout, we did not observe any significant effect of the HIIE modality on RER during the 2‐h recovery period. From a qualitative standpoint, both running and cycling elicited comparable post‐exercise substrate oxidation profiles, with similar CHOox and FATox rates (percentages and grams). As expected, FATox during recovery was higher than at rest for both modalities (Dupuit et al., 2020), reinforcing the potential of HIIE, regardless of the modality, to promote FM reduction.

Another factor influencing FM loss could be a reduction in appetite and EI. It is well known that high‐intensity exercise induces a transient decrease in appetite (Hu et al., 2022) which may contribute to lower EI. In our study, appetite scores and their subcomponents, assessed by VAS, showed similar patterns in both exercise modalities, suggesting that exercise intensity, rather than modality, plays a more significant role in regulating appetite and the desire to eat (Hu et al., 2022). These findings are consistent with a previous work in healthy young men that found no difference in perceived hunger after HIIE performed by running or cycling (Wasse et al., 2013). Furthermore, the lack of a modality effect on the 24‐h EI and macronutrient distribution is in line with the observed appetite responses.

While the findings of this pilot study provide valuable insights, some limitations should be acknowledged. First, the relatively small sample size may restrict the generalizability of the results. However, the within‐subject crossover design, in which each participant acted as their own control, helped to reduce interindividual variability and strengthened the reliability of comparisons. Additionally, post‐exercise oxygen consumption (EPOC) was only measured during the first 2 h of recovery. While this time frame captures the most metabolically active phase of EPOC, it does not allow assessing longer‐lasting effects that may occur after this time window. Some studies suggested that low‐grade elevations in EE and substrate oxidation can persist for several hours post‐exercise, particularly after high‐intensity efforts. Therefore, potential differences in the late‐phase EPOC response between running and cycling may have been overlooked, which could have implications for the total EE of each modality. Another limitation is that our study included only men. This design allowed us to reduce variability, but it prevents that direct generalizability of the findings to women, given the known sex differences in physiological and metabolic responses to exercise (Boisseau & Isacco, 2022). Therefore, similar studies in female populations would be of particular interest, especially when considering their hormonal status (menopausal vs. non‐menopausal, contraceptive use, and menstrual cycle phases).

### 4.1 Conclusion

This pilot study investigated the effects of acute HIIE, performed by cycling or running, on parameters related to FM reduction. Although RER was higher during cycling, both modalities induced similar effects on ${\dot{V}}_{O_{2}}$ and fat oxidation during the 2‐h recovery period, as well as on the total 24‐h energy and macronutrient intake. These findings suggest that, when EE is matched, running and cycling HIIE produce comparable short‐term metabolic effects and dietary intake patterns. Therefore, the choice of modality may be based on individual preference, physical condition or logistical constraints, potentially enhancing adherence in weight management programmes.

## AUTHOR CONTRIBUTIONS

Annaëlle Couvert was a PhD student who designed and supervised the different exercise sessions and wrote the first and subsequent drafts of the article under Nathalie Boisseau's guidance. Duane Beraud helped to supervise exercise sessions with Annaëlle Couvert Claire Morel was the physician who oversaw the medical aspects of the study. Bruno Pereira was responsible for statistical analyses. Mélanie Rance was a coinvestigator, assisted with the study design, and was involved in drafting the manuscript. Nathalie Boisseau conceived the study idea, was responsible for the overall study design and for monitoring data collection. Eric Doré, Vincent Martin and Antonio Herbert Lancha Junior critically reviewed the results and contributed to the final manuscript. All authors have read and approved the final version of this manuscript and agree to be accountable for all aspects of the work in ensuring that questions related to the accuracy or integrity of any part of the work are appropriately investigated and resolved. All persons designated as authors ualify for authorship, and all those who qualify for authorship are listed.

## CONFLICT OF INTEREST

The authors report no conflicts of interest.

## FUNDING INFORMATION

The authors declare no specific funding for this work.

## Supporting information



Supplementary Table 1. Acute responses during the HIIE‐RUN and HIIE‐BIKE sessions (*n* = 12).
Supplementary Table 2. Comparison of acute physiological and behavioral responses between HIIE‐RUN and HIIE‐BIKE (*n* = 12).

## Data Availability

Data available upon request.
