# Supplementary material for: Acute metabolic responses to high‐intensity interval training in men with overweight or obesity: Does the exercise modality matter?
Source: Exp Physiol. 2025 Dec 2;111(3):904–15. doi: 10.1113/EP093045 (PMC12949090; doi:10.1113/EP093045)
Supplement: Supplementary file 1 — Supplementary Table 1. Acute responses during the HIIE‐RUN and HIIE‐BIKE sessions (n = 12). Supplementary Table 2. Comparison of acute physiological and behavioral responses between HIIE‐RUN and HIIE‐BIKE (n = 12). [file EPH-111-904-s001.docx]

**Supplementary Table 1**: Acute responses during the HIIE-RUN and HIIE-BIKE sessions (n = 12).

|  |  | **HIIE-RUN** | |  | **HIIE-BIKE** | |  | **Repeated-measures ANOVA** | | | | | | |
| --- | --- | --- | --- | --- | --- | --- | --- | --- | --- | --- | --- | --- | --- | --- |
|  |  | **MEAN ± SD** | **95% CI** |  | **MEAN ± SD** | **95% CI** |  | **p-value** | | |  | ***η²*** | | |
|  |  |  |  |  |  |  |  | **T** | **M** | **T x M** |  | **T** | **M** | **T x M** |
| **V̇O_2_**  **(L.min^-1^)** | Rest | 0.33 ± 0.04 | [0.31 ; 0.36] |  | 0.33 ± 0.04 | [0.30 ; 0.35] |  | **<0.0001** | 0.143 | **0.002** |  | 0.969 | 0.000 | 0.001 |
|  | 33% HIIE | 2.22 ± 0.24 | [2.08 ; 2.37] |  | 2.26 ± 0.26 | [2.10 ; 2.43] |  |  |  |  |  |  |  |  |
|  | 66% HIIE | 2.35 ± 0.23 | [2.20 ; 2.50] |  | 2.29 ± 0.29 | [2.10 ; 2.47] |  |  |  |  |  |  |  |  |
|  | 100% HIIE | 2.41 ± 0.26 | [2.24 ; 2.58] |  | 2.30 ± 0.32 | [2.09 ; 2.50] |  |  |  |  |  |  |  |  |
|  | + 30 min | 0.41 ± 0.05 | [0.37 ; 0.44] |  | 0.41 ± 0.06 | [0.37 ; 0.45] |  |  |  |  |  |  |  |  |
|  | + 60 min | 0.40 ± 0.08 | [0.35 ; 0.45] |  | 0.38 ± 0.06 | [0.35 ; 0.42] |  |  |  |  |  |  |  |  |
|  | + 90 min | 0.37 ± 0.06 | [0.33 ; 0.41] |  | 0.38 ± 0.04 | [0.35 ; 0.41] |  |  |  |  |  |  |  |  |
|  | + 120 min | 0.41 ± 0.09 | [0.35 ; 0.46] |  | 0.40 ± 0.08 | [0.35 ; 0.45] |  |  |  |  |  |  |  |  |
| **RER** | Rest | 0.85 ± 0.04 | [0.83 ; 0.88] |  | 0.88 ± 0.04 | [0.86 ; 0.91] |  | **<0.0001** | **0.006** | **<0.0001** |  | 0.711 | 0.041 | 0.028 |
|  | 33% HIIE | 0.95 ± 0.05 | [0.92 ; 0.98] |  | 1.04 ± 0.04 | [1.01 ; 1.06] |  |  |  |  |  |  |  |  |
|  | 66% HIIE | 0.94 ± 0.04 | [0.92 ; 0.97] |  | 1.00 ± 0.04 | [0.98 ; 1.03] |  |  |  |  |  |  |  |  |
|  | 100% HIIE | 0.94 ± 0.03 | [0.92 ; 0.96] |  | 0.99 ± 0.04 | [0.96 ; 1.01] |  |  |  |  |  |  |  |  |
|  | + 30 min | 0.81 ± 0.05 | [0.78 ; 0.85] |  | 0.80 ± 0.04 | [0.78 ; 0.83] |  |  |  |  |  |  |  |  |
|  | + 60 min | 0.81 ± 0.05 | [0.79 ; 0.84] |  | 0.83 ± 0.04 | [0.81 ; 0.85] |  |  |  |  |  |  |  |  |
|  | + 90 min | 0.83 ± 0.04 | [0.81 ; 0.86] |  | 0.85 ± 0.04 | [0.82 ; 0.88] |  |  |  |  |  |  |  |  |
|  | + 120 min | 0.83 ± 0.04 | [0.80 ; 0.85] |  | 0.86 ± 0.04 | [0.83 ; 0.88] |  |  |  |  |  |  |  |  |
| **FATox**  **(g.min^-1^)** | Rest | 0.08 ± 0.02 | [0.07 ; 0.10] |  | 0.06 ± 0.03 | [0.05 ; 0.08] |  | **<0.0001** | 0.243 | **0.048** |  | 0.249 | 0.027 | 0.023 |
|  | + 30 min | 0.13 ± 0.04 | [0.10 ; 0.16] |  | 0.13 ± 0.03 | [0.12 ; 0.15] |  |  |  |  |  |  |  |  |
|  | + 60 min | 0.12 ± 0.05 | [0.10 ; 0.15] |  | 0.11 ± 0.02 | [0.09 ; 0.12] |  |  |  |  |  |  |  |  |
|  | + 90 min | 0.10 ± 0.03 | [0.08 ; 0.12] |  | 0.09 ± 0.02 | [0.08 ; 0.11] |  |  |  |  |  |  |  |  |
|  | + 120 min | 0.12 ± 0.04 | [0.09 ; 0.14] |  | 0.09 ± 0.03 | [0.08 ; 0.11] |  |  |  |  |  |  |  |  |
| **CHOox**  **(g.min^-1^)** | Rest | 0.25 ± 0.07 | [0.20 ; 0.30] |  | 0.29 ± 0.08 | [0.24 ; 0.34] |  | **0.003** | 0.195 | 0.205 |  | 0.087 | 0.027 | 0.019 |
|  | + 30 min | 0.20 ± 0.09 | [0.15 ; 0.26] |  | 0.19 ± 0.08 | [0.14 ; 0.24] |  |  |  |  |  |  |  |  |
|  | + 60 min | 0.20 ± 0.07 | [0.16 ; 0.25] |  | 0.23 ± 0.09 | [0.17 ; 0.29] |  |  |  |  |  |  |  |  |
|  | + 90 min | 0.22 ± 0.08 | [0.17 ; 0.27] |  | 0.25 ± 0.08 | [0.21 ; 0.30] |  |  |  |  |  |  |  |  |
|  | + 120 min | 0.23 ± 0.09 | [0.17 ; 0.29] |  | 0.29 ± 0.11 | [0.22 ; 0.36] |  |  |  |  |  |  |  |  |
| **FATox**  **(%)** | Rest | 48.5 ± 12.2 | [40.7 ; 56.2] |  | 38.6 ± 14.5 | [29.4 ; 47.8] |  | **<0.0001** | 0.219 | **0.014** |  | 0.201 | 0.028 | 0.027 |
|  | + 30 min | 62.2 ± 17.7 | [51.0 ; 73.4] |  | 66.2 ± 13.2 | [57.7 ; 74.6] |  |  |  |  |  |  |  |  |
|  | + 60 min | 62.0 ± 14.4 | [52.9 ; 71.2] |  | 57.0 ± 12.4 | [49.1 ; 64.8] |  |  |  |  |  |  |  |  |
|  | + 90 min | 55.3 ± 14.0 | [46.4 ; 64.1] |  | 50.3 ± 13.4 | [41.8 ; 58.9] |  |  |  |  |  |  |  |  |
|  | + 120 min | 58.1 ± 14.4 | [49.0 ; 67.3] |  | 47.9 ± 13.1 | [39.5 ; 56.2] |  |  |  |  |  |  |  |  |
| **CHOox**  **(%)** | Rest | 51.6 ± 12.2 | [43.8 ; 59.3] |  | 61.4 ± 14.5 | [52.2 ; 70.6] |  | **<0.0001** | 0.219 | **0.014** |  | 0.201 | 0.028 | 0.027 |
|  | + 30 min | 37.8 ± 17.7 | [26.6 ; 49.0] |  | 33.8 ± 13.2 | [25.4 ; 42.3] |  |  |  |  |  |  |  |  |
|  | + 60 min | 38.0 ± 14.4 | [28.8 ; 47.1] |  | 43.0 ± 12.4 | [35.2 ; 50.9] |  |  |  |  |  |  |  |  |
|  | + 90 min | 44.7 ± 14.0 | [35.9 ; 53.6] |  | 49.7 ± 13.4 | [41.1 ; 58.2] |  |  |  |  |  |  |  |  |
|  | + 120 min | 41.9 ± 14.4 | [32.7 ; 51.0] |  | 52.1 ± 13.1 | [43.8 ; 60.5] |  |  |  |  |  |  |  |  |
| **RPE** | 0% HIIE | 1.5 ± 0.8 | [0.9 ; 2.1] |  | 1.4 ± 0.9 | [0.8 ; 2.1] |  | **<0.0001** | 0.001 | **<0.0001** |  | 0.614 | 0.024 | 0.025 |
|  | 33% HIIE | 2.8 ± 1.1 | [2.1 ; 3.5] |  | 3.2 ± 0.9 | [2.6 ; 3.8] |  |  |  |  |  |  |  |  |
|  | 66% HIIE | 3.9 ± 1.2 | [3.1 ; 4.6] |  | 4.4 ± 1.3 | [3.5 ; 5.2] |  |  |  |  |  |  |  |  |
|  | 100% HIIE | 4.7 ± 1.4 | [3.8 ; 5.7] |  | 5.7 ± 1.7 | [4.6 ; 6.8] |  |  |  |  |  |  |  |  |
| **Desire to eat (mm)** | Pre-HIIE | 40.9 ± 32.3 | [20.4 ; 61.4] |  | 40.7 ± 32.7 | [19.9 ; 61.5] |  | **<0.0001** | 0.603 | 0.911 |  | 0.152 | 0.002 | 0.001 |
|  | Post-HIIE | 33.6 ± 25.8 | [17.2 ; 50.0] |  | 31.8 ± 26.6 | [15.0 ; 48.7] |  |  |  |  |  |  |  |  |
|  | +60 min | 54.1 ± 28.4 | [36.1 ; 72.2] |  | 49.6 ± 24.7 | [33.9 ; 65.3] |  |  |  |  |  |  |  |  |
|  | +120 min | 65.9 ± 28.0 | [48.1 ; 83.7] |  | 60.7 ± 27.8 | [43.0 ; 78.3] |  |  |  |  |  |  |  |  |
| **Hunger (mm)** | Pre-HIIE | 42.0 ± 33.4 | [20.8 ; 63.2] |  | 45.2 ± 36.5 | [22.0 ; 68.4] |  | **0.0001** | 0.807 | 0.897 |  | 0.121 | 0.000 | 0.001 |
|  | Post-HIIE | 35.0 ± 30.2 | [15.9 ; 54.2] |  | 37.3 ± 32.4 | [16.7 ; 57.8] |  |  |  |  |  |  |  |  |
|  | +60 min | 57.0 ± 27.5 | [39.5 ; 74.4] |  | 56.8 ± 26.4 | [40.0 ; 73.6] |  |  |  |  |  |  |  |  |
|  | +120 min | 64.2 ± 30.1 | [45.1 ; 83.3] |  | 63.2 ± 25.5 | [47.0 ; 79.4] |  |  |  |  |  |  |  |  |
| **Fullness (mm)** | Pre-HIIE | 31.5 ± 29.8 | [12.5 ; 50.4] |  | 37.3 ± 32.6 | [16.6 ; 57.9] |  | **0.002** | 0.607 | 0.497 |  | 0.062 | 0.001 | 0.004 |
|  | Post-HIIE | 35.6 ± 27.5 | [18.2 ; 53.1] |  | 36.6 ± 32.3 | [16.1 ; 57.1] |  |  |  |  |  |  |  |  |
|  | +60 min | 24.2 ± 19.6 | [11.7 ; 36.7] |  | 25.3 ± 20.7 | [12.2 ; 38.5] |  |  |  |  |  |  |  |  |
|  | +120 min | 23.0 ± 20.8 | [9.8 ; 36.2] |  | 19.6 ± 12.5 | [11.6 ; 27.6] |  |  |  |  |  |  |  |  |
| **PFC**  **(mm)** | Pre-HIIE | 66.3 ± 28.5 | [48.2 ; 84.4] |  | 63.8 ± 25.7 | [47.4 ; 80.1] |  | 0.051 | 0 .733 | 0.634 |  | 0.044 | 0.002 | 0.004 |
|  | Post-HIIE | 62.9 ± 25.6 | [46.7 ; 79.2] |  | 56.0 ± 29.8 | [37.1 ; 74.9] |  |  |  |  |  |  |  |  |
|  | +60 min | 69.8 ± 18.4 | [58.1 ; 81.5] |  | 70.2 ± 16.9 | [59.4 ; 80.9] |  |  |  |  |  |  |  |  |
|  | +120 min | 71.6 ± 20.6 | [58.5 ; 84.7] |  | 72.4 ± 19.8 | [59.9 ; 85.0] |  |  |  |  |  |  |  |  |
| **Appetite score** | Pre-HIIE | 54.4 ± 25.9 | [38.0 ; 70.9] |  | 53.1 ± 27.4 | [35.7 ; 70.5] |  | **<0.0001** | 0.746 | 0.995 |  | 0.132 | 0.001 | 0.000 |
|  | Post-HIIE | 49.0 ± 22.0 | [35.0 ; 63.0] |  | 47.1 ± 27.0 | [30.0 ; 64.3] |  |  |  |  |  |  |  |  |
|  | +60 min | 64.2 ± 18.4 | [52.5 ; 75.8] |  | 62.8 ± 17.7 | [51.6 ; 74.0] |  |  |  |  |  |  |  |  |
|  | +120 min | 69.7 ± 18.3 | [58.1 ; 81.3] |  | 69.2 ± 18.7 | [57.3 ; 81.0] |  |  |  |  |  |  |  |  |

RER: Respiratory exchange ratio; FATox: Fat oxidation; CHOox: Carbohydrate oxidation; RPE: Rate of Perceived Exertion; PFC: Perspective of Food Consumption.

T, time effect; M, modality effect; T x M, modality–time interaction

**Supplementary Table 2**: Comparison of acute physiological and behavioral responses between HIIE-RUN and HIIE-BIKE (n = 12).

|  | HIIE-RUN | |  | HIIE-BIKE | |  | Wilcoxon test |
| --- | --- | --- | --- | --- | --- | --- | --- |
|  | MEAN ± SD | CI 95% |  | MEAN ± SD | CI 95% |  | p-value |
| 2h-recovery V̇O_2_ (L.min^-1^) | 0.41 ± 0.04 | [0.39 ; 0.44] |  | 0.41 ± 0.04 | [0.39 ; 0.44] |  | 0.937 |
| 2h-recovery EE (kcal) | 240.9 ± 25.3 | [225 ; 257] |  | 241.7 ± 22.0 | [228 ; 256] |  | 0.754 |
| 2h-recovery FAT ox (g.min^-1^) | 0.12 ± 0.03 | [0.10 ; 0.14] |  | 0.11 ± 0.02 | [0.09 ; 0.12] |  | 0.388 |
| 2h-recovery CHO ox (g.min^-1^) | 0.25 ± 0.07 | [0.21 ; 0.29] |  | 0.27 ± 0.07 | [0.23 ; 0.31] |  | 0.209 |
| 2h-recovery FAT ox (g) | 13.8 ± 3.7 | [11.4 ; 16.1] |  | 12.8 ± 2.7 | [11.1 ; 14.5] |  | 0.388 |
| 2h-recovery CHO ox (g) | 30.3 ± 7.9 | [25.3 ; 35.3] |  | 33.3 ± 8.6 | [27.9 ; 38.8] |  | 0.209 |
| 2h-recovery FAT ox (%) | 56.5 ± 12.5 | [48.6 ; 64.5] |  | 54.1 ± 10.8 | [47.2 ; 60.9] |  | 0.531 |
| 2h-recovery CHO ox (%) | 43.5 ± 12.5 | [35.5 ; 51.4] |  | 45.9 ± 10.8 | [39.1 ; 52.8] |  | 0.531 |
|  |  |  |  |  |  |  |  |
| Desire to eat (AUC) | 7256 ± 3665 | [4927 ; 9584] |  | 6731.8 ± 3429.4 | [4553 ; 8911] |  | 0.117 |
| Hunger (AUC) | 7444 ± 3821 | [5017 ; 9871] |  | 7531.8 ± 3889.9 | [5060 ; 10003] |  | 0.583 |
| Fullness (AUC) | 4146± 3241 | [2087 ; 6205] |  | 4222.6 ± 3255.4 | [2154 ; 6291] |  | 0.875 |
| PFC (AUC) | 10020 ± 3010 | [8107 ; 11932] |  | 9694.9 ± 2730.9 | [7960 ; 11430] |  | 0.239 |
| Appetite score (AUC) | 8836 ± 2709 | [7114 ; 10557] |  | 8616.8 ± 2825.7 | [6821 ; 10412] |  | 0.117 |

AUC: area under the curve; FAT ox: Fat oxidation; CHO ox: Carbohydrate Oxidation; EE: Energy Expenditure; PFC: Perspective of Food Consumption.
